# Medicinal Cannabis and Synthetic Cannabinoid Use

**DOI:** 10.3390/medicina56090453

**Published:** 2020-09-07

**Authors:** Simona Pichini, Alfredo Fabrizio Lo Faro, Francesco Paolo Busardò, Raffaele Giorgetti

**Affiliations:** 1 Analytical Pharmacotoxicology Unit, National Centre on Addiction and Doping, Istituto Superiore di Sanità V.Le Regina Elena 299, 00161 Rome, Italy; simona.pichini@iss.it; 2 Department of Excellence of Biomedical Sciences and Public Health, “Politecnica delle Marche” University of Ancona, Via Tronto 10/a, 60126 Ancona, Italy; fabriziolofaro09@gmail.com (A.F.L.F); r.giorgetti@univpm.it (R.G.)

**Keywords:** synthetic cannabinoids, urine, liquid chromatography, high-resolution mass spectrometry, gas chromatography-mass spectrometry

## Abstract

Cannabis products have been used for centuries by humans for recreational and medical purposes. Resent research, proposed the promising therapeutic potential of cannabis and related cannabinoids for a wide range of medical conditions, including psychiatric and neurological diseases. This Special Issue presents the latest updates on medicinal cannabis and synthetic cannabinoids pharmacology, toxicology and new analytical methods to identify and quantify these compounds in conventional and non-conventional biological matrices. Moreover, it provides current data regarding their adverse effects, safety, application for medical purposes and their harmful effects.

According to the World Health Organization, cannabis is the most cultivated, trafficked, seized and consumed drug of abuse worldwide [1]. At the same time, its medical use in several diseases is growing in popularity. Many chronic pathologies, including neurogenic pain, for which no effective treatment has been found to date, are benefiting from medical cannabis [2,3].

In the early 2000s, Synthetic Cannabinoids (SCs), molecules that mimic the effects of Δ^9^-tetraidrocannabinol (THC), designed to act on the endocannabinoids system, appeared in the illegal market. Furthermore, in the last few years, in countries where cannabis legalization has not taken place, such as Italy, some manufacturers have started producing and selling the so called “light cannabis” [3]. This last product consisted of dried flowering tops containing the main psychoactive principle of cannabis, i.e., THC, at concentrations lower than 0.2%, and a variable concentration of cannabidiol (CBD), another major non-psychotropic phytocannabinoid shown to reduce anxiety or insomnia [4].

In this Special Issue, attention has been given to different neurological aspects following the use of phytocannabinoids and SCs, with particular consideration given to their prevalence, consumption and potential health threats, also addressing some updates on SCs.

Mammana et al. started this Special Issue with a research article on the medical use of cannabis products. The authors described the pharmacological properties of CBD alone and in combination with another phytocannabinoid cannabigerol (CBG), and their potential clinical applications, especially in neurodegenerative diseases. Based upon their results, the authors confirmed CBD’s and CBG’s anti-inflammatory, anti-oxidant and anti-apoptotic properties and suggested their use for the treatment of many neurodegenerative diseases [5].

This topic was also addressed by Poyatos et al., who reviewed the available data on cannabis and THC pharmacokinetics (PK) after oral administration in humans. The authors discussed the safety and effectiveness of oral formulations as adjuvant treatment or alternative therapy for treatment-resistant patients. Although several formulations of cannabis have been recommended for medical use, data on cannabinoids’ PK after oral administration are scarce and limited to particular pharmaceutical forms, such us tablets and capsules. In conclusion, additional investigations were advocated to make safer preparations available, avoiding the adverse effects of accidental THC overdosing [6].

Brunetti et al. examined the most common cannabis-based pharmaceutical products and their medical indication (e.g., route of administration, posology), and provided guidance to assist medical practitioners in their decision-making process to prescribe and manage medical cannabis use [7].

Tamba et al. assessed the current data on the toxicity of SC through in vivo and in vitro trials, their pharmacological properties (including PK and pharmacodynamics (PD)), and their identification in animal and human biological fluids. The authors discussed different strategies to improve the bioavailability of SC and their application in pain management with minimal adverse effects, providing a better understanding of SC as useful and alternative analgesic drugs [8].

In this concern, Orsolini et al. discussed the potential use of medical cannabis and SC as a therapeutic approach in patients with **post-traumatic stress disorder** (PTSD) when the first-line treatment (i.e., antidepressant and anxiolytic medication) was ineffective. The reported experimental data demonstrated promising results, mainly in reducing nightmares and sleep disorders due to PTSD, even if several adverse effects such us seizures, respiratory depression and hyperthermia were shown following daily SC use. The authors suggested to focus research efforts on the safety and tolerability of SC and cannabis products in the treatment of psychiatric disorders [9].

In their original investigation, Tejedor-Cabrera et al. explored the risk of drug abuse associated with marijuana/hashish and alcohol use was assessed in young nursing students. The authors reported that the consumption of cannabis-derived products and alcohol was higher among the youngest male student (<25 years of age) and correlated with personal and social consequences such as the inability to stop drinking once started or to remember what happened while drinking. The authors concluded that it is necessary to implement a set of effective strategies to prevent or change drug- and alcohol-related behaviors to healthier ones [10].

Regarding the prevalence and the risk of cardiovascular and cerebrovascular disease in young cannabis users (18–39 years), Desai et al. indicated increasing trends in hospitalizations without concomitant abuse of other drugs, and found that marijuana use was associated with a higher risk of cardiovascular events [11].

The rapid growing popularity of new SC on the darknet and the constant synthesis of new substances have become a challenge for forensic laboratories. This analytical challenge involves not only the large range of compounds and metabolites to detect, but also **to discern the type of biological specimens (conventional and non-conventional matrices) to be investigated** [12]. Pellegrini et al. proposed a screening method for the quantification of three SC (JWH-122, JWH-210 and UR-144) and their respective metabolites JWH-122 N-(4-hydroxypentyl), JWH-122 N-(5-hydroxypentyl), JWH-210 N-(4-hydroxypentyl), JWH-210 N-(5-hydroxypentyl), UR-144 N-(4-hydroxypentyl) and UR-144 N-(5-hydroxypentyl) in urine using a fast sample extraction and two different analytical techniques: ultra-high-performance liquid chromatography–high-resolution mass spectrometry (UHPLC-HRMS) and high-sensitivity gas chromatography–mass spectrometry (GC-MS) [13].

We hope that this issue will be contribute to the advancement of knowledge currently available in the field of medicinal cannabis and SCs and the global issue that cannabis and its derived products have generated since their appearance on the market.
